# Battery ingestion: an unusual cause of mediastinitis

**DOI:** 10.1590/S1806-37132014000500016

**Published:** 2014

**Authors:** Rosana Souza Rodrigues, Fátima Aparecida Ferreira Figueiredo, César Augusto Amorim, Gláucia Zanetti, Edson Marchiori

**Affiliations:** Serviço de Radiodiagnóstico, Hospital Universitário Clementino Fraga Filho, Universidade Federal do Rio de Janeiro; e Médica, Instituto D’Or de Pesquisa e Educação, Rio de Janeiro, RJ, Brasil; Instituto D’Or de Pesquisa e Educação; e Médica, Departamento de Medicina Interna, Universidade Estadual do Rio de Janeiro, Rio de Janeiro, RJ, Brasil; Instituto D’Or de Pesquisa e Educação; e Médico, Departamento de Medicina Clínica, Universidade Federal do Rio de Janeiro, Rio de Janeiro, RJ, Brasil; Programa de Pós Graduação em Radiologia, Universidade Federal do Rio de Janeiro, Rio de Janeiro, RJ, Brasil; Universidade Federal Fluminense, Niterói, RJ, Brasil; e Professor Associado, Universidade Federal do Rio de Janeiro, Rio de Janeiro, RJ, Brasil

## To the Editor:

An 18-month-old boy with dry cough and fever was brought to the emergency department. His mother reported the onset of the symptoms approximately 3 days prior. She reported no nausea, vomiting, refusal to eat, or other symptoms. Physical examination revealed no abnormalities.

**Figure 1:**
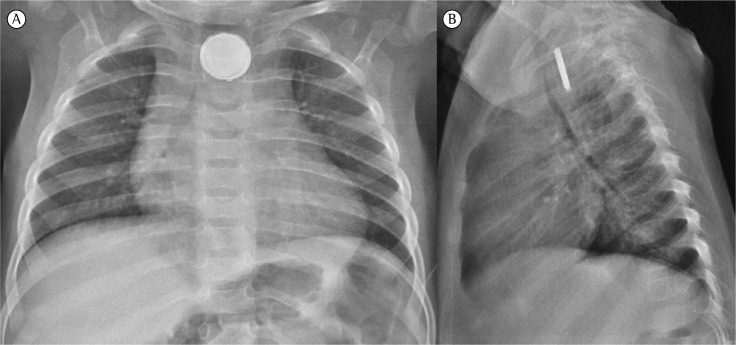
In A, an anteroposterior chest X-ray revealing a circular radiopaque shadow with a peripheral double rim or a halo effect, and, in B, a lateral X-ray showing a step-off, which allowed the diagnosis of a button battery lodged in the esophagus.

Chest X-rays demonstrated a round, opaque object lodged at the upper esophagus. The identification of a circular radiopaque shadow with a peripheral double rim or a halo effect on an anteroposterior (AP) X-ray (Figure 1A) and a step-off on the lateral view (Figure 1B) allowed the diagnosis of button battery (BB) ingestion.

**Figure 2:**
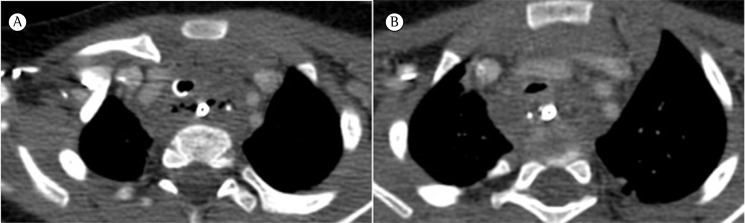
CT scans showing upper mediastinum widening with areas of low attenuation surrounding the trachea and supra-aortic arteries and veins, as well as extraluminal gas and metallic fragments (remains of the foreign body), suggestive of esophageal perforation and mediastinitis.

Esophagoscopy showed a corroded 20-mm-diameter lithium BB lodged in the esophagus, with corrosive injury of the mucosa. The foreign body was retrieved 80 h after the ingestion. CT scans showed foci of material with high density posterior to the trachea (remains of the foreign body) and signs of esophageal perforation and mediastinitis (Figure 2).

Immediate surgical consultation was done, and conservative treatment was decided (observation, antibiotics and nasogastric tube feeding). After 30 days of hospitalization, follow-up esophagoscopy demonstrated complete lesion closure with no sign of stenosis.

A BB lodged in the esophagus can cause severe tissue damage and delayed complications, such as esophageal perforation, tracheoesophageal fistulas, mediastinitis, and death.^(^ 1 ^-^ 4 ^)^ Misdiagnoses frequently occur when ingested batteries are misidentified on X-rays as other objects, particularly coins. However, subtle differences exist in the radiographic features of BBs and coins. A halo of reduced density is present around the circumference of a BB (double rim or halo effect) on an AP X-ray, and a step-off can be observed on the lateral view.

A battery lodged in the esophagus must be treated as a medical emergency because of its rapid corrosive action; a BB may cause serious burns in just two hours.^(^ 1 ^-^ 4 ^)^ Patients with a battery in the esophagus may be asymptomatic initially. Endoscopic removal is preferred because it allows direct visualization of tissue injury.^(^ 1 ^)^
