# Supplementary material for: Battery ingestion: an unusual cause of mediastinitis
Source: J Bras Pneumol. 2014 Sep-Oct;40(5):582–3. doi: 10.1590/S1806-37132014000500016 (PMC4263341; doi:10.1590/S1806-37132014000500016)
Supplement: Translation

# Translation: Ingestão de bateria: uma causa incomum de mediastinite

**Authors:** Rosana Souza Rodrigues, Fátima Aparecida Ferreira Figueiredo, César Augusto Amorim, Gláucia Zanetti, Edson Marchiori

**Affiliations:** Serviço de Radiodiagnóstico, Hospital Universitário Clementino Fraga Filho, Universidade Federal do Rio de Janeiro; e Médica, Instituto D’Or de Pesquisa e Educação, Rio de Janeiro, RJ, Brasil; Instituto D’Or de Pesquisa e Educação; e Médica, Departamento de Medicina Interna, Universidade Estadual do Rio de Janeiro, Rio de Janeiro, RJ, Brasil; Instituto D’Or de Pesquisa e Educação; e Médico, Departamento de Medicina Clínica, Universidade Federal do Rio de Janeiro, Rio de Janeiro, RJ, Brasil; Programa de Pós Graduação em Radiologia, Universidade Federal do Rio de Janeiro, Rio de Janeiro, RJ, Brasil; Universidade Federal Fluminense, Niterói, RJ, Brasil; e Professor Associado, Universidade Federal do Rio de Janeiro, Rio de Janeiro, RJ, Brasil

## Ao Editor:

Um menino de 18 meses de idade, apresentando tosse seca e febre, foi levado ao serviço de emergência. A mãe relatava início dos sintomas há cerca de 3 dias. Negava náuseas, vômitos, recusa em comer e outros sintomas. Ao exame físico, não foi observada nenhuma alteração.

Radiografias de tórax demonstraram a presença de um objeto redondo e opaco alojado no esôfago superior. A identificação de uma sombra radiopaca circular com dupla borda periférica ou efeito auréola na radiografia em incidência anteroposterior (AP; Figura 1A) e de uma sombra em declive na radiografia em perfil (Figura 1B) permitiu o diagnóstico de ingestão de bateria botão (BB).

**Figura 1:** Em A, radiografia de tórax em incidência anteroposterior revelando uma sombra radiopaca circular com dupla borda periférica ou efeito auréola, e, em B, radiografia em perfil mostrando uma sombra em declive, o que permitiu o diagnóstico de bateria botão alojada no esôfago.

A esofagoscopia mostrou a presença de uma BB de lítio corroída de 20 mm de diâmetro alojada no esôfago, com lesão corrosiva da mucosa. O corpo estranho foi removido 80 h após a ingestão. A TC mostrou focos de material com alta densidade localizados posteriormente à traqueia (restos de corpo estranho) e sinais de perfuração esofágica e mediastinite (Figura 2). 

**Figura 2:** TC mostrando alargamento do mediastino superior com áreas de baixa atenuação em torno da traqueia e das artérias supra-aórticas e veias, bem como gás extraluminal e fragmentos metálicos (restos de corpo estranho), sugestivos de perfuração esofágica e mediastinite.

Realizada consulta cirúrgica imediata, decidiu-se por tratamento conservador (observação, antibióticos e alimentação por sonda nasogástrica). Após 30 dias de internação, a esofagoscopia de controle demonstrou fechamento completo da lesão, sem nenhum sinal de estenose.

Uma BB alojada no esôfago pode causar graves danos teciduais e complicações tardias, como perfuração esofágica, fístulas traqueoesofágicas, mediastinite e óbito.^(^ 1 ^-^ 4 ^)^ Diagnósticos errôneos ocorrem com frequência quando as baterias ingeridas são erroneamente identificadas nas radiografias como sendo outros objetos, especialmente moedas. Porém, existem diferenças sutis nas características radiográficas das BB e das moedas. Uma auréola de densidade reduzida se faz presente em torno da circunferência de uma BB (dupla borda ou efeito auréola) na radiografia em incidência AP, e uma imagem em declive pode ser observada na radiografia em perfil.

Uma bateria alojada no esôfago deve ser tratada como emergência médica em razão de sua rápida ação corrosiva; uma BB pode causar queimaduras graves em apenas duas horas.^(^ 1 ^-^ 4 ^)^ Pacientes com bateria no esôfago podem ser assintomáticos inicialmente. Prefere-se a remoção endoscópica porque ela permite a visualização direta da lesão tecidual.^(^ 1 ^)^
